# Supplementary material for: Perspectives of people experiencing homelessness with recent non-fatal street drug overdose on the Pharmacist and Homeless Outreach Engagement and Non-medical Independent prescribing Rx (PHOENIx) intervention
Source: PLoS One. 2024 May 13;19(5):e0302988. doi: 10.1371/journal.pone.0302988 (PMC11090330; doi:10.1371/journal.pone.0302988)
Supplement: S1 File — (DOC) [file pone.0302988.s001.doc]

**S1. Appendix. Logic model of PHOENIx intervention for people experiencing homelessness with a recent non-fatal overdose**

**Problems**

Overwhelmed by multiple complex health- and social care needs and self-management; lack of tailored generalist outreach service to meet health needs; leading to low uptake of preventative care; re-traumatising living conditions; undiagnosed health conditions; perceived fragmentation of services; drug use to block traumatic memories; overdose; early mortality

**Inputs**

Independent prescriber clinical pharmacist and third sector worker engagement on outreach; health- and social care (benefits, housing and social prescribing)

**Intervention/ activities**

PHOENIx health- and social care intervention.

Weekly contacts for 6 to 9 months.

Assessment of physical, mental health, addictions, housing, benefits and social activities.

Agreed priority areas for help.

Medicines prescribing by pharmacist where necessary, direct treatment e.g. wounds.

Referrals to health and social care.

Rapid referral to substance misuse teams/ED

**Short-term outcomes**

Improved engagement with health and social care.

Assessment of symptoms.

Therapeutic relationship and trust with PHOENIx.

Immediate treatment of presenting conditions e.g. wounds.

Support self-management.

Prescribing.

Immediate urgent referral to health.

Assessment for benefits, housing and social prescribing.

Reduced workload and burden of illness.

Reduced pain and disability.

Reduced emergency treatment.

**Medium-term outcomes**

Initiation of treatment for additional conditions e.g. addictions, mental health, physical health.

Reduced impact of care burden.

Maximising benefits of housing application.

Accessing social prescribing opportunities.

Increased engagement with health- and social care services.

**Longer-term outcomes**

Improvement in surrogate markers of disease.

Reduced overdoses.

Reduction in unscheduled ED visits.

Reduction in unscheduled GP or out of hours visits.

Wider social impacts.

Reduced costs.

**Potential moderators of effect:** social circumstances, age, gender, underlying health conditions including addiction/mental health conditions. **Potential mediators of effect:** intervention fidelity, Normalisation Process Theory constructs (Coherence – making sense, Cognitive Participation – engagement and intervention, Collective Action – confidence/acceptability of intervention/workability, Reflexive Monitoring – appraisal/individual specification and reconfiguration). **ED:** emergency department. **GP:** general practitioner. **Cross cutting themes:** trauma informed workforce, comprehensive care, continuity of care, coordinated care.
