# Supplementary material for: Perspectives of people experiencing homelessness with recent non-fatal street drug overdose on the Pharmacist and Homeless Outreach Engagement and Non-medical Independent prescribing Rx (PHOENIx) intervention
Source: PLoS One. 2024 May 13;19(5):e0302988. doi: 10.1371/journal.pone.0302988 (PMC11090330; doi:10.1371/journal.pone.0302988)
Supplement: Attachment — Submitted filename: PlosOneMan_Ed.doc [file pone.0302988.s003.doc]

**Perspectives of people experiencing homelessness with recent non-fatal street drug overdose, on the Pharmacist and Homeless Outreach Engagement and Non-medical Independent prescribing Rx (PHOENIx) intervention**

Natalia Farmer^1*¶^, Andrew McPherson^2¶^, Jim Thomson^3¶^, Richard Lowrie^2, 4¶^

^1^School of Social Work, Glasgow Caledonian University, Glasgow, United Kingdom

^2^Pharmacy Services, NHS Greater Glasgow and Clyde, Glasgow, United Kingdom

^3^We See You Project, Glasgow, United Kingdom

^4^Centre for Homelessness and Inclusion Health, University of Edinburgh, Edinburgh, United Kingdom

*Corresponding author

E-mail: [Natalia.farmer@gcu.ac.uk](mailto:Natalia.farmer@gcu.ac.uk) (NF)

¶These authors contributed equally to this work.

**Abstract**

**Introduction**

In Scotland, a third of all deaths of people experiencing homelessness (PExH) are street-drug-related, and less than half of their multiple physical- and mental health conditions are treated. New, holistic interventions are required to address these health inequalities. PHOENIx (Pharmacist Homeless Outreach Engagement and Non-medical Independent prescribing Rx) is delivered on outreach by National Health Service (NHS) pharmacist independent prescribers in partnership with third sector homelessness charity workers. The pharmacist addresses health problems, while the third sector worker focusses on welfare benefits, housing and social prescribing. PHOENIx aims to reduce emergency department visits and overdoses in PExH by supporting their health and social care needs. We describe participant’s perspectives of PHOENIx in the context of a pilot randomised controlled trial.

**Methods**

This qualitative study aims to understand experiences of the PHOENIx intervention by 20 of the 62 participants recruited into the active arm of a pilot randomised controlled trial (RCT) conducted between September 2021 and June 2022, in Glasgow, Scotland, United Kingdom. Semi-structured in-person interviews explored participants’ evaluation of the intervention. In this study, the four components (*coherence*, *cognitive participation*, *collective action*, *reflexive monitoring*) of the Normalisation Process Theory (NPT) framework underpinned data collection and analyses. Ethical approval for the study was granted by the East of Scotland National Health Service Research Ethics Committee 01.

**Results**

We identified four themes that were interpreted within the NPT framework that describe participant evaluation of the PHOENIx intervention: differentiating the intervention from usual care (*coherence*), embedding connection and consistency in practice (*cognitive participation*), implementation of practical and emotional operational work (*collective action*), and lack of power and a commitment to long-term support (*reflexive monitoring*). Participants successfully engaged with the intervention due to perceived benefits often lacking in their usual care provision. Facilitators for participant motivation included the relationship-based work created by the PHOENIx team, providing retention by means of continuous investment. This included operational work to fulfil both the practical and emotional needs of participants. Barriers included concern regarding power imbalances within the sector, a lack of long-term support and the impact of the intervention concluding.

**Conclusions**

The findings identify and describe participants’ evaluations of the PHOENIx intervention. NPT is a theoretical framework facilitating understanding of experiences, highlighting both facilitators and barriers to sustained engagement and investment. Our findings inform future developments regarding a subsequent definitive RCT of PHOENIx, despite challenges brought about by the changing micropolitics within the local health and social care partnership in Glasgow.

**Keywords**: Homelessness, chronic disease, opioid addiction, primary health care, randomised controlled trial, public health, polydrug use, drug-related death, normalisation process theory

**Introduction**

People experiencing homelessness (PExH) have multiple unmet health- and social care needs [1], contributing to premature mortality [2]. PHOENIx (Pharmacist Homeless Outreach Engagement and Non-medical Independent prescribing Rx), a collaborative and complex health- and social care intervention, aims to address these needs through weekly assertive outreach by National Health Service (NHS) pharmacists and third sector homelessness caseworkers. By providing intensive and holistic health- and social care support and persistent follow-up, a key objective is to reduce overdose and emergency service utilisation. Participants are visited via outreach and seen by the same pair of PHOENIx workers (pharmacist and third sector worker) at least once per week, with priorities set by the person experiencing homelessness and tackled gradually, in succession. As well as providing immediate medical care, health assessment and medication prescribing, PHOENIx offers practical support e.g. advocacy, support for application for welfare benefits, clothing, phones. Working collaboratively with key stakeholders, such as primary care general practitioners (GPs), emergency department staff, community alcohol- and drug recovery services, and social care services, previous qualitative research has shown that PHOENIx may improve health [3]. Moreover, initial quantitative studies carried out on the PHOENIx intervention highlights that it can have a positive impact on the health-related quality of life of individuals [4].

The PHOENIx intervention is set against a backdrop of a third of all deaths in Scotland in people experiencing homelessness are drug-related [5]. There is, however, a paucity of research on interventions to tackle the intersecting issues of homelessness and problem drug use [6], despite homelessness itself having a bi-directional causal pathway with illicit drug use and overdose [7]. Across Scotland, drug-related death (DRD) rates are increasing and remain one of the highest in the world. In Glasgow, over 70% of PExH have problem street-drug use [8] and, while most are receiving care from Alcohol and Drug Recovery Services (ADRS), including daily prescribed opiate replacement therapy, they continue to overdose. There is a need for interventions that prevent premature mortality, frequent emergency department visits by people experiencing homelessness, and overdoses [9]. People experiencing homelessness also have a higher incidence of post-traumatic stress disorder, major depression and other chronic health conditions, including blood borne viruses, contributing to increased readmission rates to hospital [10]. Permanent, supportive accommodation can impact positively on mental health and wellbeing in people experiencing homelessness [11], but is only part solution with the main critical attribute of health-seeking behaviour of people experiencing homelessness being distrust in healthcare from stigma-associated behaviours by health providers [12]. PHOENIx aims to address DRDs in a subgroup of those at highest risk of DRD (homeless and recent non-fatal overdose) through outreach-based assessment and treatment of a wide range of health determinants, and interventions prioritised by the patient. [1,13]

There is an urgent need for evaluation of innovative approaches to address the public health crisis of drug-related deaths in Scotland. Definitive RCTs are the gold standard approach to demonstrating the efficacy of complex interventions and, prior to that, feasibility studies and pilot RCTs are recommended to confirm recruitment, retention, data collection, intervention fidelity, and barriers and facilitators to real world implementation [14]. A feasibility study of the PHOENIx intervention was conducted and suggested merit in progressing to a pilot RCT [4]. Subsequently, the PHOENIx after overdose randomised controlled trial (RCT) recruited 128 PExH with recent non-fatal street drug overdose, allocating 62 participants to PHOENIx intervention (weekly outreach visits by pharmacist and third sector worker in addition to care as usual, for 10 months), and 66 participants to care as usual [1,13,15]. Parallel process evaluations are becoming increasingly important in healthcare RCTs, because they can help to contextualise the delivery and receipt of public health interventions [16]. They are often recognised as an integral feature of a randomised controlled trial of a complex intervention [17] and can assist with interpretation of outcomes [18] due to inherent difficulties in defining or developing a new intervention [19]. Moreover, they help to provide insight into the “black box” of the intervention by investigating the discrete perceptions of participants [20]. Previous evaluations of PHOENIx have explored stakeholders’ views, highlighting the ability of the intervention to provide a ‘sticky’, ‘informal’ and ‘flexible’ service that provides support to overcome barriers to health- and social care for hard-to-reach populations that fall through the gaps in care delivery [3]. However, in this study, evaluation focused on how the intervention was perceived by participants experiencing homelessness with a recent non-fatal overdose and in the context of a pilot RCT, as opposed to stakeholders delivering the intervention. This approach was chosen to investigate specific mechanisms of impact, such as participants’ responses and interactions with PHOENIx [21], in order to facilitate careful consideration in regards to intervention improvement and development from a patient-led perspective. A logic model of the PHOENIx intervention for people experiencing homelessness is illustrated in S1 Fig.

**Methods**

**Study design**

This qualitative study was part of a larger pilot randomised controlled trial (RCT). Recruitment to the pilot RCT started on the 11^th^ of May 2021 and was completed on the 1^st^ of September 2021. Informed written consent to participate in the embedded qualitative study was obtained by independent researcher A.M. as part of consent to participate in the RCT quantitative study.

We used Normalisation Process Theory (NPT) to understand and describe how participants in the active part of the RCT experienced and evaluated the PHOENIx intervention. NPT has roots in science and technology studies and can be seen as the antithesis of actor-network theory because of the assertion of explanation over description [22]. A systematic review of NPT for process evaluation of complex interventions found that it provides an important theoretical framework to support comprehension and implementation of interventions in transformational healthcare [23] and can be useful to understand the implementation of complex interventions in health care systems [24]. NPT offers a robust framework that focuses upon the processes and operational aspects of what individuals and teams do in order to implement an intervention [25]. This involves the use of four NPT constructs outlined in Table 1: *coherence* (sense-making work), *cognitive participation* (relational work), *collective action* (operational work), and *reflexive monitoring* (appraisal work). Together, these constructs help foster understanding in regards to how an intervention is implemented, operationalised and experienced within health- and social care settings. NPT has been successfully utilised within qualitative research to further understand complex pharmacist-led interventions for medication safety in primary care [26] and general practice responses to opioid prescribing [27]. In this study, we aimed to use NPT to aid data collection and analyses in order to identify and understand how people experiencing homelessness with recent non-fatal overdose experienced and evaluated the PHOENIx intervention.

**Table 1. NPT construct in association with the implementation of PHOENIx**

| **NPT Construct** |  |
| --- | --- |
| **Coherence** | Sense-making work to understand the possibilities of an intervention.  What is the purpose of PHOENIx? |
| **Cognitive participation** | Relational work that builds a community of practice around an intervention.  What promotes participation with PHOENIx? |
| **Collective action** | Operational work that people enact to make an intervention function.  How do participants interact with PHOENIx to make them work? |
| **Reflexive monitoring** | Appraisal work where people assess how a new practice affects them and others.  How do participants appraise PHOENIx? |

**Participants**

Eligible participants were aged 18 years old or older, defined by the European Typology of Homelessness and Housing Exclusion (ETHOS) as homeless (roofless, houseless, insecure accommodation) with at least one non-fatal street-drug overdose, due to illicit drug use in the 6 months prior to recruitment to the study. Twenty participants were then purposively sampled by independent researchers A.M and N.F from the intervention arm of the PHOENIx pilot RCT [13], using a heterogenous sampling technique in order to ensure maximum variation and capture a range of perspectives across gender, age, accommodation and number of prior overdoses. Participant characteristics are outlined in Table 2.

**Table 2. Participant characteristics**

| **Sex**  Male  Female | 13 (65%)  7 (35%) |
| --- | --- |
| **Age**  Mean (SD) | 45 (8.7) |
| **Number of overdoses (past 6 months)**  Median (range) | 2 (1–30) |
| **Accommodation**  Supported  Unsupported  Hospital | 6 (30%)  12 (60%)  2 (10%) |
| **Opiate Substitute Therapy**  Methadone  Buprenorphine  Nil | 18 (90%)  1 (5%)  1 (5%) |
| **Methadone dose (mg/ml)**  Mean (SD) | 90 (22.8) |
| **Received diazepam prescription**  Yes  No | 1 (5%)  19 (95%) |
| **Naloxone**  Possess Naloxone  Knows how to use it | 12 (60%)  17 (85%) |
| **Alcohol and Drug Recovery Service (ADRS)**  Generic ADRS  Homeless ADRS  Not known to ADRS | 13 (65%)  6 (30%)  1 (5%) |

SD=Standard deviation

**Data collection**

Twenty face-to-face semi-structured interviews were conducted by N.F., an academic qualified social worker with experience in qualitative research with marginalised individuals and communities and not involved in the delivery of the trial intervention. Interviews were conducted between November 2021 and January 2022 in-person in emergency- or hostel accommodation or in a private space within a homeless charity (Simon Community Scotland) drop-in hub. Interview questions were guided by the NPT framework and lasted a median duration of 40 minutes, with a range from 30 to 60 minutes. The interview template was designed with feedback from individuals from a third sector charity with lived- and living experience of homelessness and overdose. This was to ensure rigour in the formulation of research questions by identifying gaps or problematic areas, such as appropriate language/terminology, and revise accordingly. The final interview template is provided in S2 Fig. Following interviews, each participant was given a pseudonym to ensure anonymity and confidentiality. Participants received a £10 shopping voucher in recognition for their participation in the interviews. Interviews were recorded using an encrypted electronic device and transcribed verbatim using an external organisation approved by the NHS.

**Data analysis**

Normalisation Process Theory (NPT) was utilised in order to gain a deeper understanding of participants’ evaluations of the PHOENix intervention. An initial reflexive thematic analysis (RTA) [28] was conducted by N.F and A.M after transcripts were entered into NVivo Version 12 software. This approach was taken in order to mitigate against simply ‘shoehorning’ [29] data into the predetermined NPT constructs. This enabled orientation with the data, generation of initial codes, developing themes and reviewing them with the team (N.F, A.M, R.L and J.T), alongside consideration of researcher positionality within knowledge production. Following this stage, data themes were mapped to a coding framework informed by the NPT constructs: *coherence* (sense-making work); *cognitive participation* (relational work); *collective action* (operational work); and *reflexive monitoring* (appraisal work). In order to ensure rigour and reliability during the process of data analysis, N.F and A.M adopted the reflexive pair method [30], in order to consider researcher positionality within knowledge production and ensure consistency in the generation of codes and developing themes. Additionally, the involvement of co-authors (R.L and J.T) provided additional insight and feedback that helped to develop iterative revisions and ensure further consistency and credibility during the interpretation and written analysis.

**Ethical approval**

The study was approved by South East Scotland National Health Research Ethics Committee 01. All participants were provided with participant information sheets and were able to ask questions about the study and use of data before providing informed written consent. All data were collected and are stored in accordance with NHS General Practice Data for Planning and Research (GPDPR) guidelines. Trial registration number: ISRCTN10585019.

**Results**

We used the consolidated criteria for reporting qualitative research (COREQ) as a framework to guide reporting of our research. Four components that promoted further understanding into how participants evaluated the implementation of PHOENIx into practice and was informed by the NPT constructs of *coherence* (what is the purpose of PHOENIx), *cognitive participation* (what promotes participation with PHOENIx), *collective action* (how do participants interact with PHOENIx) and *reflexive monitoring* (how do participants appraise and rate their care provided by PHOENIx). The four components of NPT and how the participants evaluated PHOENIx alongside this are outlined in Table 3. In this section we explore the perceptions of participants across the components to consider how they evaluated the intervention. Specific attention is given to our aim of understanding factors that either promoted or inhibited the incorporation of PHOENIx in their everyday lives. In turn, identifying any gaps or issues that would require the intervention to be resigned or improved in future delivery.

**Table 3. Overview of themes identified through the NPT constructs**

| Coherence | Cognitive Participation | Collective Action | Reflexive Monitoring |
| --- | --- | --- | --- |
| Differentiating the intervention from usual care | Embedding connection and consistency in practice | Implementation of practical and emotional operational work | Lack of power and a commitment to long-term support |

**Coherence: differentiating the PHOENIx intervention from usual care**

The first NPT construct, *coherence* refers to the sense-making work that participants went through and shared in interviews regarding their understanding of the purpose and possibilities of the PHOENIx intervention. Specifically, participants revealed an important element of this process rested upon their perceptions of the management of drug overdose in general and the roles of other different health- and social care providers. Within the data, contrasting perceptions of their usual care in comparison to the PHOENIx intervention were consistently expressed by participants. Provision provided by the PHOENIx team was differentiated from other health- and social care teams, with participants stating their unhappiness with Alcohol and Drugs Recovery Services (ADRS) in contrast to their satisfaction with the intervention. Many described their usual care as inadequate as opposed to PHOENIx, which was understood to be beneficial because it provided aspects of care and support lacking from other health- and social care providers.

For example, participants expressed negative opinions in regards to the realities of ADRS management and significant frustration in terms of their ability to successfully manage their drug use and health problems. There was an overall sense of being ‘dismissed’ when visiting their addictions teams alongside a distinct lack of help:

You go in [laughs] they see you, “How are you, how’s your drug use been this week, are you feeling all right, how’s your injecting site?” They don't look at you or anything like that, they just ask you questions. “Right, do you think you need up 10ml or another 20ml on your methadone? No, right, here’s script for 2 weeks or whatever.” That's not help. (Sean)

I mean everything is coming from myself, they’ve not helped, I mean I can’t say that they have because they’ve not, and I’m not going to sit here and say that they’ve done something that they haven’t. (Clare)

On the other hand, participants significantly differentiated PHOENIx from the usual care they received and, overall, participants provided positive feedback of PHOENIx and in terms of the management of drug- and health problems, the purpose of the intervention was perceived as providing a service that was often missing from other teams. Hence, the benefits of the intervention were clear, as the below two quotes highlight:

I found [PHOENIx staff] more supportive than my drug worker, you know what I mean? She [ADRS worker] was increasing me instead of decreasing me [Methadone]. Then she wasn’t doing the call she should be doing, calling me to see if everything was okay. How you doing with your methadone? She wasn’t doing anything like that. It was [PHOENIx staff] who was contacting me. (Daniel)

The [ADRS team] are piss, but what they [PHOENIx staff] do is brilliant, you know, it's like night and day with the [ADRS team] and they [PHOENIx staff] they [PHOENIx staff] says, “I'm going to do something.” and they do it. (Brian)

Overall, the intervention was perceived as beneficial, because it was seen to be providing essential support regarding the management of drug overdose in areas of health- and social care that participants stated was absent.

**Cognitive participation: embedding connection and consistency in practice**

*Cognitive participation* refers to the relational work that is required and involves engaging and motivating individuals around a new intervention. A component of this construct is ‘enrolment’, which entails the complex work that is involved in getting individuals to ‘buy in’ to new practices or processes. It influences both motivation and commitment, and is an important aspect in regards to ensuring successful participation. The findings revealed that participants highly valued both the (1) connection and (2) consistency that members of the PHOENIx team provided in order to build and sustain a relationship and actively engage individuals with the intervention. In conjunction, these two aspects created the motivation that was required for individuals to both initially engage with a new service and to continue their sustained involvement with the team on an ongoing basis.

Participants frequently highlighted the relational connection that they had with members of the PHOENIx team, stating that the genuine and caring approach taken enabled them to feel willing and motivated to spend time with the team. The non-judgemental approach shown by PHOENIx staff was vital in order to create trust and confidence with participants, placing an emphasis on authenticity and sincerity. Consequently, this related to participants feeling able to trust the team, which in turn created feelings of comfort and care, as highlighted by the below quotes:

I’ve been brought up in care and I’ve had a really hard life so I can see through a bullshitter, I can see through somebody who genuinely cares or are just doing their job. I can tell if they actually give a shit or not. (Anna)

I’ve got a good relationship with them. Very supportive, and they care, they really do care. I trust them. And it is that trust. They’ve been there for me, they’ve been supportive, and that’s what I needed or else I think I’d have done something daft a long time ago. (Sarah)

Comfortable. Every time we were with them, they made us feel comfortable. They didn't speak down to me. I just felt we were on an even keel with them, do you know what I mean? Aye, just being comfortable and you can just tell when somebody is on your side. (Caroline)

Secondly, consistency was emphasised as an integral aspect that enabled participants to sustain continued participation and motivation during the trial intervention. Importantly, this was related to the level of meaningful connection that has been established with members of the team and their ability to create long-lasting relationships that were consistent. The following are examples that highlight the important of consistency and reliability within relational work that helped to empower and sustain connections.

They were the only workers really consistent. My care manager, my other worker, I don’t know her as well as I know [PHOENIx team], if you know what I mean. Any time I was in trouble I would go to one of them. I can just tell that they care. (Anna)

Just seeing [PHOENIx team] on a weekly basis is beneficial for me […] That’s a goal for me, seeing people like that motivates me to stay sober. It's embarrassing when you have to see them on the Wednesday and you took something you shouldn't have, you’re like pure wounded, pure defeated. That feeling of being defeated is powerful and I don't want that in my life anymore. (Brian)

I think they're brilliant because you’re not going to get penalised by missing an appointment […] they're not going to be on your back, they're not going to walk out on you. A couple of times I got really depressed, thinking, I'm not letting them in, I don't want to see anybody and I thought, they're not going to want to see us again and they came out. (Caroline)

In general, participants described the connection that they developed with members of the PHOENIx team as an essential component required for their participation with the intervention. Given the extensive stigma and discrimination encountered by those who use drugs and are homelessness, for the intervention to be successful, this particular aspect is crucial in terms of both initial ‘buy-in’ and continued investment and retention. Therefore, establishing that the personal attitudes and skill set of the PHOENIx team in their ability to build an authentic relationship with participants is an essential aspect of the intervention in terms of securing trust and confidence.

**Collective Action: implementation of practical and emotional operational work**

*Relational integration* is a component of the *collective action* construct that refers to the knowledge work enacted by individuals that enables confidence and accountability within a new intervention. This aspect of PHOENIx was emphasised significantly and discussed in detail during the interviews. It provided clarity in regards to the work that the PHOENIx team operationalised that was most meaningful and useful from the perspectives of participants. The findings focused upon two sub-themes that participants identified as most valuable for them in terms of managing the ongoing stress burdens they encountered in their everyday lives struggling to meet their health- and social needs. Specifically, this referred to the interactional and integrated outreach work that the pharmacists and third sector caseworkers conducted in fulfilling both the (1) practical needs and (2) emotional needs of participants.

The practical outreach the team provided was a key factor that the majority of participants highlighted as most useful and highly valued, because it reduced the individual stress burdens experienced by the cohort. This was especially crucial given the known discrimination and barriers encountered by PExH when attempting to access health- and social care within the context of homelessness and drug use. The knowledge base and advocacy skills of the PHOENIx team in regards to addressing immediate healthcare needs during outreach visits was reported by all participants. This included the ability of pharmacists to treat some health conditions e.g. wound care, assess severity of health conditions e.g. respiratory problems, and prescribe for all health conditions except problem drug use (as this was the exclusive role of Alcohol and Drug Recovery Services) on outreach, as described within the below quotes:

They got me an appointment with the hospital, they've rearranged appointments for me at the [acute hospital] because I missed that, all because I've been taking seizures for the last couple of years, a couple of them are drug-induced seizures and the [PHOENIx team] rearranged the appointment for me because I forgot about it, because I didn't have it written down. (Brian)

They helped my legs. I’ve had eight abscesses in my legs now. I’ve had all sorts of blood clots. If they didn’t get me the help I was getting at the time, I’d probably would have lost that one. They found out I had a heart murmur. I think they recognised that I’ve got a lot of internal stuff as well I’m having to deal with. (Lee)

Participants also stressed the importance they gained from the intervention in regards to the advocacy that the team provided with social care, which in turn alleviated the stress burden associated with navigating various social welfare systems. Again, the knowledge and skills of third sector staff to provide support with Universal Credit, Personal Independence Payments (PIP) and housing applications was frequently reported, and these systems without support elevated participants anxiety and stress.

We never had Wi-Fi, never had a computer, never had a phone. Sometimes, things were building up on my phone, things we had to do, it was just all building up and [PHOENIx team] would come out and just ask us what we needed done and he would sit there for ages on the computer doing the electricity thing and I’ve got [PHOENIx team] over here during a PIP form for me, and it's taking him like 45 minutes just to get through and then he's on the phone for an hour. Practical and I appreciated it. (Caroline)

They [PHOENix team] would address each new thing. They can open doors no other service can. They got me into a place when I was really desperate, cold, absolutely ill, you know what I mean? They were always trying to get me accommodation. Somewhere safe to stay, you know? (Lee)

In addition, the emotional support that participants reported they received throughout the intervention was consistently highlighted as a vital aspect that served to reduce stress levels by providing hope and humanity within the context of a highly stigmatised environment. The relational element of outreach visits emerged as an important quality and participants described an increase in confidence with each regular visit, helping them to maintain their motivation, with something to look forward to.

They come in and help and stuff, and just having somebody to talk to, they keep my spirits up, do you know what I mean? And then that plants that seed in my head, not to touch drugs. Every morning I wake up I’m like, right, no drugs today. (Sarah)

They gave me a wee bit of hope, do you know what I mean? Not easy at first, but they made me feel comfortable and didn’t make me feel belittled or anything, you know? The way they speak to you, they treat you like a human being rather than see you like a junkie. So, it gives you hope, do you know what I mean? (Lee)

It’s actually took the stress off me. I feel less stressful. I feel a lot better. (Daniel)

Together, the combined practical and emotional qualities embedded within PHOENIx outreach visits fostered a level of confidence within participants that mitigated against the increased stress burdens many encountered in their attempts to navigate health- and social care systems. This enabled individuals to receive immediate, accessible and potentially life-saving healthcare and social advocacy that they would otherwise be unable to access.

**Reflexive monitoring: lack of power and a commitment to long-term support**

*Reflexive monitoring,* the appraisal work that is crucial to understand how the PHOENIx intervention was assessed was limited at the individual level, with many participants struggling to retain engagement towards the end of the interview and, notably, repetition in answers was evident. However, when asked if they would recommend the PHOENIx intervention, the overall response was highly positive and all had or would recommend to others due to the characteristics of the intervention explored in this paper. While no specific negative issues arose, there were changes and anxieties that may lead to the reconfiguration of the intervention, or, at least, an opportunity to redefine or modify communication with participants at the outset.

An initial aspect that participants suggested would have been beneficial during the intervention involved the incorporation of regular food parcels during outreach visits. Additionally, some participants were keen to see members of the PHOENIx team have more ‘power’ and felt that staff regularly had their ‘hands tied’ and were unable to enact as much change as desired in various areas, such as mental health provision:

The workers to get a wee bit more say in things. That’s about it. If they did get more of a say in things, they could do a wee bit more for addicts. I think you should be given that wee bit more power of things. If you were given that wee bit more power there would be a lot more people’s lives saved. (Lee)

They tried to get me some counselling, and they got knocked back. They got me listed for the [third sector organisation], so I’m listed for counselling, but there’s no date yet, but I know I need counselling. (Brian)

Furthermore, it is important to recognise that towards the end of the interview, participants strongly expressed their anxiety that the PHOENIx intervention and outreach visits were not ongoing. Consequently, this seemed to have a negative impact on individuals, with many aware that once visits and the trial ended, the commitment to long-term support would end. As the below highlights:

Because my journey is just starting and I am scared I will relapse. I would like to come out of the treatment centre and still have their support, because it’s still going to be fresh. I’m scared in case I relapse and nobody is there to help me. (Anna)

I feel as if I’m going to lose contact with [PHOENIx team]. And eventually not see them again. That’ll just knock me right back into depression again. (Matthew)

**Discussion**

The purpose of this study was to gain an understanding into how participants experiencing homelessness with a recent non-fatal overdose evaluated PHOENIx in order to identify factors that either promoted or inhibited the incorporation of the intervention in their everyday lives. Informed by NPT, analysis revealed several factors within the four constructs that participants emphasised as critical to the success of the intervention, alongside gaps in implementation that could be enhanced or resigned for future delivery.

The sense-making work (*coherence****)*** needed to understand the possibilities of an intervention was an important aspect in participants understanding of the purpose of PHOENIx. As previous research has demonstrated, the intervention is highly effective in terms of engaging with people experiencing homelessness who encounter barriers when attempting to access health- and social care, leading to many falling through the gaps [3]. However, these findings provide further understanding in regards to why initial engagement with PHOENix is so successful in the management of drug overdose. Participants overwhelmingly reported that the intervention was crucial for them due to a consistent sense of being failed within other health- and social care services. [31]. There was significant differentiation between the intervention and the usual care that they received, creating a clear purpose and benefit in PHOENIx. The management of drug overdose from other service providers, particularly ADRS teams was described negatively, leading to the importance of PHOENIx in providing essential support that many stated was lacking elsewhere.

For the intervention implementation to be successfully sustainable, a crucial aspect that participants emphasised was the relational work the PHOENIx team effectively created (*cognitive participation*). This was related to how engaged and motivated participants felt working with the team during the trial period. Guidance suggests the importance of ‘buy-in’ with a new intervention [32]. However, within a context marred by previous relational injury such as stigma [33], this study underscored the critical necessity for strong and supportive relationships, whereby non-judgemental characteristics within the team are central to creating and maintaining participation. This was evidenced in terms of the ability of the PHOENIx staff to create what participants described as a genuine, authentic and sincere approach. Furthermore, participants emphasised that two specific components, ‘connection’ and ‘consistency’ were key in the success of building relationships with the team, which, in turn, fostered trust, confidence and continued reliability.

PHOENIx is provided through a combination of NHS and third sector individuals handpicked with the requisite human relational skills and non-judgemental approach to care. In terms of how participants experienced the intervention, this collaborative skill-set was crucial and highlighted to be the most useful aspect of PHOENIx for several reasons, due to the implementation of both practical and emotional operational work (*collective action*). Practically, participants described a reduction in their daily stress burdens, as the team were able to immediately treat some health conditions and organise or rearrange health- and social care appointments. Previous research has highlighted the importance measuring treatment burden for clinical trials [34]. Our findings support this and provide more detail in the context of homelessness and drug overdose, and also aligns well with emerging signs of delayed overdoses, emergency department visits and hospitalisations in the intervention arm of the pilot randomised controlled trial, but these returned to pre-PHOENIx after the intervention visits ended. Moreover, participants stressed that challenges navigating various social welfare systems, in particular Universal Credit, PIP and housing applications created additional burdens that increased levels of anxiety. For them, the advocacy and knowledge that third sector members of the team provided were highly valued, which is most relevant given the increasing extent of digital exclusion among people living in areas of socioeconomic disadvantage and the ubiquitous role that digital technologies play in health- and social care systems [35]. Participants further acknowledged that the emotional support provided by the team not only provided much needed hope within the stigmatised context of homelessness and drug use [33], but it also increased their motivation and sustained engagement and retention within the intervention. Given that research has gaps within service delivery in regards to mental health and counselling within the context of addiction service [36], it is worth noting how stronger mental health provision might be incorporated into the future intervention design.

Overall, participants evaluated the PHOENIx intervention highly (*reflexive monitoring*), with all participants stating that they would recommend the intervention and expressed a desire for outreach visits to continue for reasons highlighted in this discussion. However, there was a sense from among participants that the trial was due to end, creating anxiety and disappointment. Recent guidance has highlighted the importance of supportive, caring and therapeutic relationships [37], yet in the context of the closure of the specialist GP service for PExH in Glasgow [38], the ability to commit to long-term support is questioned. Furthermore, when asked what participants would like to see change, evaluations pointed towards structural issues within service delivery, as many reported that they would like to see the PHOENIx team have extended permissions to manage mental health and problem drug use on outreach.

It is important to note that this study is not set in a vacuum, but within dynamic political and structural environments. Financial reductions to homelessness services and increasing numbers of homelessness presentations purports that there is an ever greater need for services to address and adapt to the consequential health- and social care shortfall. Finally, study findings are particularly apposite given the recent decision by the local health- and social care partnership to discontinue the specialist homelessness GP service and the PHOENIx service in Glasgow, despite available evidence suggesting merit in retaining both.

**Conclusions**

This research has utilised NPT to reveal important factors for successful implementation of the PHOENIx intervention from the perspectives of participants. It is important to acknowledge that this study has limitations, in that it explored participant experiences of the intervention and did not include wider stakeholders. Previous studies on PHOENIx have brokered stakeholder observations and while these studies did not look at PHOENIx in the context of a pilot RCT, they did nevertheless describe the intervention. Unlike the current study, previous qualitative work did not involve participants who were, by design, at higher risk of street-drug overdose. In this study, our aim was to focus on participant evaluations [17, 39] in order to inform changes to the intervention in use, and in turn, prevent overdoses and worsening health. Given our key aim, a significant strength within this study involved the inclusion of those with lived- and living experience of homelessness and non-fatal overdose within the methodological design and analysis of data. Our robust approach ensured that feedback was sought at each phase of the research process to ensure rigour and trustworthiness. Consequently, this developed our understanding of what factors are needed to ensure engagement and continued retention from the perspectives of participants. Powerful components of the intervention were the connection and consistency fostered by the PHOENIx team in order to create both initial ‘buy-in’ and continued engagement. Findings suggest this rests on the relational skill set of the team in order to create authentic and meaningful relationships with participants. Practical and emotional operational work provided by both pharmacists and third sector caseworks was significantly valued in order to reduce stress levels of participants and foster much needed hope within a highly stigmatised context. Aspects that could optimise support include the incorporation of mental health provision and food parcels within the delivery of the intervention.

**References**

1. Lowrie R, McPherson A, Mair FS, Stock K, Jones C, Maguire D, et al. Baseline characteristics of people experiencing homelessness with a recent drug overdose in the PHOENIx pilot randomised controlled trial. Harm Reduct J. 2023;20(46). doi: [/10.1186/s12954-023-00771-4](https://doi.org/10.1186/s12954-023-00771-4).
2. Aldridge RW, Menezes D, Lewer D, Cornes M, Evans H, Blackburn RM, et al. Causes of death among homeless people: a population-based cross-sectional study of linked hospitalisation and mortality data in England. Wellcome Open Res. 2019 Mar 11;4:49. [doi: 10.12688/wellcomeopenres.15151.1](https://doi.org/10.12688/wellcomeopenres.15151.1).
3. Johnsen S, Cuthill F, Blenkinsopp J. Outreach-based clinical pharmacist prescribing input into the healthcare of people experiencing homelessness: a qualitative investigation. BMC Health Serv Res. 2021;21(7). doi: [10.1186/s12913-020-06013-8](https://doi.org/10.1186/s12913-020-06013-8).
4. Lowrie R, Stock K, Lucey S, Knapp M, Williamson A, Montgomery M, et al. Pharmacist led homeless outreach engagement and non-medical independent prescribing (Rx) (PHOENIx) intervention for people experiencing homelessness: a non-randomised feasibility study. Int J Equity Health. 2021;20(1):19. doi: 10.1186/s12939-020-01337-7.
5. National Records for Scotland. Homeless deaths in 2022. Edinburgh: Crown Copyright; 2023. Available from: <https://www.nrscotland.gov.uk/files//statistics/homeless-deaths/22/homeless-deaths-22-report.pdf>
6. Miler JA, Carver H, Masterton W, Parkes T, Maden M, Jones L, et al. What treatment and services are effective for people who are homeless and use drugs? A systematic ‘review of reviews’. PLoS ONE. 2021;16(7):e0254729. [doi: 10.1371/journal.pone.0254729](https://doi.org/10.1371/journal.pone.0254729).
7. Doran KM, Fockele CE, Maguire M. Overdose and Homelessness – Why we need to talk about housing. JAMA Netw Open. 2022 Jan 4;5(1):e2142685. doi: 10.1001/jamanetworkopen.2021.42685. PMID: 34994799.
8. Queen AB, Lowrie R, Richardson J, Williamson AE. Multimorbidity, disadvantage, and patient engagement within a specialist homeless health service in the UK: an in-depth study of general practice data. BJGP Open. 2017;1(3):1-10. doi: 10.3399/ bjgpopen17X100941.
9. Vohra N, Paudyal V, Price MJ. Homelessness and the use of Emergency Department as a source of healthcare: a systematic review. Int J Emerg Med. 20233;15(32). [doi: 10.1186/s12245-022-00435-3](https://doi.org/10.1186/s12245-022-00435-3).
10. Subedi K, Acharya B, Ghimire S. Factors Associated With Hospital Readmission Among Patients Experiencing Homelessness, American Journal of Preventive Medicine. 2022;6(3):Pages 362-370. [doi: 10.1016/j.amepre.2022.02.004](https://doi.org/10.1016/j.amepre.2022.02.004).
11. Onapa H, Sharpley CF, Bitsika V, McMillan ME, MacLure K, Smith L, et al. The physical and mental health effects of housing homeless people: A systematic review. Health & Social Care in the Community. 2022;30:448–468. [doi: 10.1111/hsc.13486](https://doi.org/10.1111/hsc.13486).
12. Becker JN, Foli KJ. Health-seeking behaviours in the homeless population: A concept analysis. Health & Social Care in the Community. 2022;30:e278– e286. [doi: 10.1111/hsc.13499](https://doi.org/10.1111/hsc.13499).
13. Lowrie R, McPherson A, Mair F, Maguire D, Paudyal V, Blair B, et al. Pharmacist and Homeless Outreach Engagement and Non-medical Independent prescribing Rx (PHOENIx): a study protocol for a pilot randomised controlled trial. BMJ Open. 2022;12:e064792. doi: 10.1136/bmjopen-2022-064792.
14. Skivington K, Matthews L, Simpson SA, Craig P, Baird J, Blazeby JM, et al. A new framework for developing and evaluating complex interventions: update of Medical Research Council guidance. BMJ. 2021;374:n2061. doi:10.1136/bmj.n2061.
15. ISRCTN. Pharmacy Homeless Outreach Engagement Non-medical Independent prescribing Rx (PHOENIx) after drug overdose for people experiencing homelessness. 2020. Available from: [doi: 10.1186/ISRCTN10585019](https://doi.org/10.1186/ISRCTN10585019).
16. Moore GF, Audrey S, Barker M, Bond L, Bonell C, Hardeman W, et al. Process evaluation of complex interventions: Medical Research Council guidance. BMJ. 2015;350:h1258. doi: 10.1136/bmj.h1258.
17. French C, Pinnock H, Forbes G, Skene I, Taylor SJC. Process evaluation within pragmatic randomised controlled trials: what is it, why is it done, and can we find it?-a systematic review. Trials. 2020 Nov 9;21(1):916. doi: 10.1186/s13063-020-04762-9.
18. Oakley A, Strange V, Bonell C, Allen E, Stephenson J. Process evaluation in randomised controlled trials of complex interventions. BMJ. 2006;332:413 doi: 10.1136/bmj.332.7538.413.
19. Campbell M, Fitzpatrick R, Haines A, Kinmouth AL, Sandercock P, Spiegelhalter D, Tyrer P. Framework for design and evaluation of complex interventions to improve health. BMJ. 2000;321:694. doi: 10.1136/bmj.321.7262.694.
20. Minary L, Trompette J, Kivits J, Cambon L, TarquinioC, Alla F. Which design to evaluate complex interventions? Toward a methodological framework through a systematic review. BMC Med Res Methodol. 2019;19(92). doi: 10.1186/s12874-019-0736-6.
21. Moore G, Audrey S, Barker M, Bond L, Bonell C, Cooper, C et al. Process evaluation in complex public health intervention studies: the need for guidance J. Epidemiol. Community Health. 2014;68:101-102. doi: 10.1136/jech-2013-202869.
22. May CR, Finch T, Ballini L, MacFarlane A, Mair F, Murray E, et al. Evaluating complex interventions and health technologies using normalization process theory: development of a simplified approach and web-enabled toolkit. BMC Health Serv Res. 2011;11(245). doi: 10.1186/1472-6963-11-245.
23. May CR, Cummings A, Girling M, Bracher M, Mair F, May CM, et al. Using Normalization Process Theory in feasibility studies and process evaluations of complex healthcare interventions: a systematic review. Implementation Sci. 2018;13(80). doi: 10.1186/s13012-018-0758-1.
24. May CR, Finch T, Mair F, Ballini L, Dowrick C, Eccles M, et al. Understanding the implementation of complex interventions in health care: the normalization process model. BMC Health Serv Res. 2007;7(148). doi: 10.1186/1472-6963-7-148.
25. Murray E, Treweek S, Pope C, MacFarlane A, Ballini L, Dowrick C,. et al. Normalisation process theory: a framework for developing, evaluating and implementing complex interventions. BMC Med. 2010;8(63). doi: [10.1186/1741-7015-8-63](https://doi.org/10.1186/1741-7015-8-63).
26. Jeffries M, Keers RN, Phipps DL, Williams R, Brown B, Avery AJ, et al. Developing a learning health system: insights from a qualitative process evaluation of a pharmacist-led electronic audit and feedback intervention to improve medication safety in primary care. PLoS One. 2018;13(10), e0205419.
27. Wood S, Foy R, Willis TA, Carder P, Johnson S, Alderson S. General practice responses to opioid prescribing feedback: a qualitative process evaluation. British Journal of General Practice. 2021;71(711):e788-e796.
28. Braun V, Clarke V. Can I use TA? Should I use TA? Should I not use TA? Comparing reflexive thematic analysis and other pattern-based qualitative analytic approaches. Counselling and Psychotherapy Research. 2021;21(1):37–47.
29. Gallacher K, Jani B, Morrison D, Macdonald S, Blane D, Erwin, P, et al. Qualitative systematic reviews of treatment burden in stroke, heart failure and diabetes-methodological challenges and solutions. BMC medical research methodology. 2013;13:1-10.
30. Gilmore S, Kenny K. Work-worlds colliding: Self-reflexivity, power and emotion in organizational ethnography. Human relations. 2015;68(1):55-78.
31. Finch D, Wilson H, Bibby J. Leave no one behind: The state of health and health inequalities in Scotland. The Health Foundation. 2023. RL: doi: 10.37829/HF-2023-HL01.
32. O'Cathain A, Croot L, Duncan E, Rousseau N, Sworn K, Turner KM, et al. Guidance on how to develop complex interventions to improve health and healthcare. BMJ Open. 2019 Aug 15;9(8):e029954. doi: 10.1136/bmjopen-2019-029954.
33. Reilly J, Williamson A. A systematic review of the effect of stigma on the health of people experiencing homelessness. Health and Social Care in the community. 2022;30(6):2128-2141.
34. Jones C, Mair FS, Williamson AE, McPherson A, Eton DT, Lowrie R. Treatment burden for people experiencing homelessness with a recent non-fatal overdose: a questionnaire study. British Journal of General Practice 2023;73(735):e728-e734. doi: <https://doi.org/10.3399/BJGP.2022.0587>.
35. Holmes H, Burgess G. Digital exclusion and poverty in the UK: How structural inequality shapes experiences of getting online, Digital Geography and Society. 2022;3. 100041, ISSN 2666-3783. doi: [10.1016/j.diggeo.2022.100041](https://doi.org/10.1016/j.diggeo.2022.100041).
36. Baingana F, al'Absi M, Becker A, . et al. Global research challenges and opportunities for mental health and substance-use disorders. Nature. 2015;527:S172–S177. [doi: 10.1038/nature16032](https://doi.org/10.1038/nature16032).
37. National Institute for Health and Social Care Excellence. Integrated health and social care for people experiencing homelessness. NICE guideline 214. 2022 Mar 16. Available from: [www.nice.org.uk/guidance/ng214](http://www.nice.org.uk/guidance/ng214)
38. McArdle H, Closure of Glasgow homeless GP service ‘very worrying’. The Herald. 2023 Apr 25. Available from: <https://www.heraldscotland.com/news/23479227.closure-glasgow-homeless-gp-service--worrying/>
39. Tjia J, Kutner JS, Ritchie CS, et al. Perceptions of statin discontinuation among patients with life-limiting illness. J Palliat Med. 2017;20:1098–103. doi: 10.1089/jpm.2016.0489.

**Supporting information**

**Supporting File 1. Logic model: PHOENIx intervention for people experiencing homelessness with a recent non-fatal overdose**

**Supporting File 2. Interview Guide**
