# Supplementary material for: Perspectives of people experiencing homelessness with recent non-fatal street drug overdose on the Pharmacist and Homeless Outreach Engagement and Non-medical Independent prescribing Rx (PHOENIx) intervention
Source: PLoS One. 2024 May 13;19(5):e0302988. doi: 10.1371/journal.pone.0302988 (PMC11090330; doi:10.1371/journal.pone.0302988)
Supplement: Attachment — Submitted filename: S2_File_Ed.doc [file pone.0302988.s005.doc]

**Supporting File 2. Interview Guide**

**Study title: PHOENIx after overdose**

Some participants will be asked to participate in a telephone qualitative interview. The interview will be semi-structured in format to encourage patients to share their experiences of the existing pathway for health- and social care follow-up after overdose, and acceptability of the PHOENIx intervention.

Questions have been developed using NPT, a framework that has been shown to help gain understanding into how new services are implemented in practice.

Participant Name: ___________________________________________________________________

Study ID: _________________________

Date & time of interview: _____________________________________________________________

Interviewer name: __________________________________________________________________

Consent form signed? YES	_🞏_

**Interview guide:**

1. Answer any questions, confirm informed consent.
2. Explain purpose of the interview.
3. Use question guide (outlined over).
4. Any further questions or comments about the resource (or participating in the trial itself)?
5. Thanks for participation.

The interview is semi-structured so the questions outlined here are a guide to the general topics/areas of interest that will be investigated.

**Coherence – what is the understanding of the management of drug overdose and the roles of different health professionals? What do they think of this PHOENIx intervention?**

a) What do you understand by the term overdose and what do you have to do to manage your drug use?
b) Which health professionals helped you manage your drug use and how well do you think they managed all your health problems?
c) What is your experience of visiting your addictions team? Did you attend any addictions appointments while participating in this study?
d) What is your experience of visiting your GP? Did you attend any GP appointments during this study?
e) What did you think about having a pharmacist/nurse and Simon Community worker (PHOENIx team) come out and help with the management of your drug use and other health problems?

**Cognitive participation – how well did you engage with the PHOENIx team?**

a) Did you feel motivated to try and work with the PHOENIx team?
b) How did you feel about having to spend time with the team?
c) Did others (e.g. family/friends/medical professionals etc.) help or hinder your participation in the trial or your use of the service? Describe how?
d) Did your views of the PHOENIx team or the trial change over time?

**Collective action – how useful was the PHOENIx intervention?**

a) How useful did you find the PHOENIx visits? What aspects of the visits did you particularly like or dislike?
b) Did the PHOENIx intervention help you manage your drug use? If yes, how? If not, why not? Did it fit in with the way you manage your drug use at the moment?
c) Did the PHOENIx visits make you more- or less likely to contact health professionals or health services? Why?
d) Did the visits make you more- or less likely to visit A&E or call an ambulance?
e) How much did you trust the advice/information provided by the PHOENIx team? Explain.
f) What did you think about the trial questionnaires you had to complete as part of the trial? Did you think any of them asked about things that are particularly important to you? If yes, which ones? If not, what would you have liked to be asked about?

**Reflexive Monitoring – overall what did you think about this trial and the help the pharmacists provided?**

a) What aspects of your health/drug use did the PHOENIx team affect most (positively/negatively)?
b) What, if anything, would you change about the help you received?
c) If someone asked you whether you would recommend the PHOENIx visits, what would you tell them and why?
d) If possible, would you like to have continued access to the PHOENIx? Why?
e) Were there any aspects of being involved in the trial that you found difficult or any procedures that you particularly disliked? If yes, what would you like to see done differently in a future large-scale trial?
f) Would you participate in a large-scale trial of this service if approached again? If not, please explain your answer.
