# Supplementary material for: Perspectives of people experiencing homelessness with recent non-fatal street drug overdose on the Pharmacist and Homeless Outreach Engagement and Non-medical Independent prescribing Rx (PHOENIx) intervention
Source: PLoS One. 2024 May 13;19(5):e0302988. doi: 10.1371/journal.pone.0302988 (PMC11090330; doi:10.1371/journal.pone.0302988)
Supplement: Attachment — Submitted filename: Response to Reviewers.doc [file pone.0302988.s006.doc]

Dear Drs Scheibe and Janse van Rensburg

All authors associated with this research would like to thank you for your professional insight and your recommended amendments to our manuscript, which enhance it and provide additional clarity for the reader. We are in a state of flux in Glasgow with a reduction in drug and alcohol funding from central and devolved governments. The service that we provide to these patients is crucial, yet the powers that control matters have decided to terminate it, alongside specialist GPs working in homeless health. We would like to point you to our recent findings publication <https://bmjpublichealth.bmj.com/content/2/1/e000219> which paints a very stark picture of addictions, services for people experiencing homelessness and drug-related deaths. Despite this, there is hope that, publications like this one, will reach the decision-makers and make them realise the dedication, commitment, psychotherapeutic skills and compassion workers in PHOENIx have and that this works for patients, and just maybe the service can see a reversal of fortunes, and we will see PHOENIx rise from the ashes and on the streets of Glasgow once more.

**Academic Editor**

1. We have checked PLOS ONE’s style requirements and amended as required (author contributions have been explored in more detail, the abstract word count now sits at 300 words, stated patient and public involvement in the study and updated the references, including the reference list.
2. Financial statement should now read;

The authors’ received funding from NHS Greater Glasgow and Clyde and the Scottish Government Drug Deaths Task Force. The funders had no role in study design, data collection and analysis, decision to publish, or presentation of the manuscript.

3. Unsure of the data sharing protocols, we wrongly provided details of data sharing for the quantitative part of our Pilot RCT. Data sharing of interviews wold not be appropriate because of the (very real) potential for identification of participants and associated sensitive data. The data availability statement should now read;

The full manuscripts from the semi-structured interviews are not available as they contain potentially identifying and sensitive participant information. Participants were not informed or requested to allow transcripts to be shared publicly.

4. We have reviewed our reference list for completion and correctness. Furthermore, we have updated it to provide further contextual evidence.

**Additional Editor Comments:**

Methods and ratio of people approached invited to participate – all people approached/invited to participate in this study. We have stated this and provided a potential rationale for the high response rate.

Table 2. Methadone dose. This has now been change to mg only.

The limitations have been expanded to include potential bias from researchers and possible mitigation, with potential influence on study findings.

We have strengthened the conclusion by providing recommendations for future research and the utility of NPT as a research method.

**Reviewer 2**

We have checked the referencing with the Vancouver referencing guide and amended it accordingly. We wish to thank Dr Janse van Rensburg with her assistance in this matter.

1. The objectives of the study are now clearly stated with bullet points.
2. The inclusion and exclusion criteria for the study has been added and a brief description of the intervention and usual care has been given with a reference for more detail on these
3. Usual care has been defined within the text and readers are directed to the findings paper of the Pilot RCT for further details.
   4.1 We have clarified where interviews were carried out and set out how we maintained privacy.

4.2 We asked participants to provide us with a pseudonym for the qualitative interviews in order to maintain privacy and confidentiality. We have stated this process in the manuscript text.

5. We have clarified the sentence; Our robust approach ensured feedback, and we have included a section on patient public involvement

6. With regards to the interview guide Line 59 mentioning only pharmacists, this should read “pharmacist and third-sector charity worker” and we have amended

7. We have clarified the issue of consent and detailed this in the manuscript

We have clarified the issue over data availability. Given that participants were interviewed and this was transcribed, it would be inappropriate for this to be public given that participant identification together with sensitive data may inadvertently occur.
